# Anatomical Correlates of Age-Related Working Memory Declines

**DOI:** 10.4061/2011/606871

**Published:** 2011-11-16

**Authors:** Evan T. Schulze, Elizabeth K. Geary, Teresa M. Susmaras, James T. Paliga, Pauline M. Maki, Deborah M. Little

**Affiliations:** ^1^Department of Neurology and Rehabilitation, University of Illinois at Chicago, Chicago, IL 60612, USA; ^2^Duke University College of Medicine, Durham, NC 27704, USA; ^3^Department of Psychology, University of Illinois at Chicago, Chicago, IL 60612, USA; ^4^Department of Psychiatry, University of Illinois at Chicago, Chicago, IL 60612, USA; ^5^Department of Anatomy & Cellular Biology, University of Illinois at Chicago, Chicago, IL 60612, USA; ^6^Department of Ophthalmology & Visual Sciences, University of Illinois at Chicago, Chicago, IL 60612, USA; ^7^Department of Veterans Affairs, VISN 17 Center of Excellence for Research on Returning War Veterans, Waco, TX 76711, USA; ^8^Texas A&M Health Sciences, College of Medicine, Bryan, TX 77807, USA

## Abstract

Aging studies consistently show a relationship between decreased gray matter volume and decreased performance on working memory tasks. Few aging studies have investigated white matter changes in relation to functional brain changes during working memory tasks. Twenty-five younger and 25 older adults underwent anatomical magnetic resonance imaging (MRI) scans to measure gray matter volume, diffusion tensor imaging (DTI) to measure fractional anisotropy (FA) as a measure of white matter integrity, and functional magnetic resonance imaging (fMRI) while performing a working memory task. Significant increases in activation (fMRI) were seen in the left dorsal and ventral lateral prefrontal cortex with increased working memory load and with increased age (older showing greater bilateral activation). Partial correlational analyses revealed that even after controlling for age, frontal FA correlated significantly with fMRI activation during performance on the working memory task. These findings highlight the importance of white matter integrity in working memory performance associated with normal aging.

## 1. Introduction

Although there is some debate about the magnitude of age-related effects on gray matter (GM) and white matter (WM), it is generally accepted that both GM and WM volumes decline with advanced age [1–3]. Furthermore, declines in certain cognitive skills are also anticipated with advanced age [4]. Examinations of cortical volume and behavior suggest a relationship between volume loss and declines in cognitive skills. In particular, fluid-intelligence skills such as working memory, believed largely mediated by frontal-subcortical structures [4–8], appear particularly susceptible to age-related changes [9–12]. Multiple neuroimaging techniques have been utilized in isolation to examine the diffuse neural networks supporting complex behaviors, but only recently have multimodal imaging techniques such as standard structural imaging, functional magnetic resonance imaging (fMRI), and diffusion tensor imaging (DTI) been utilized concurrently to elucidate the relationship between volume loss, white matter integrity, and declines in cognitive functions associated with healthy aging. 

Standard magnetic resonance imaging (MRI) structural studies have historically examined structure-function relationships in aging with an emphasis on volumetric alterations. While debate exists regarding the relationship between volume and function, these prior studies have reported correlations between frontal gray matter (GM) density and behavioral measures of executive function [13, 14], frontal white matter (WM) integrity and executive function, and overall prefrontal cortex volume and problem solving, working memory, and processing speed [15]. 

Diffusion tensor imaging (DTI) is a relatively newer neuroimaging modality, which allows for the examination of the integrity and directionality of white matter tracts. WM integrity is measured using factional anisotropy (FA) values, which are calculated based on the directionality of the diffusion of water. Increased isotropic diffusion and low directionality indicate demyelination or axonal loss and are correlated with a low FA value (near 0). With increased age, a loss of integrity as indicated by a decreased FA value is observed in WM fiber tracts and throughout the whole brain [16–19]. As with studies of GM and WM volumetric changes, differential decreases in FA are often observed within the frontal lobe WM tracts, including the superior frontal gyrus [20–24]. 

Unlike standard structural MRI, fMRI uses the properties of blood flow and oxygen concentration (Blood oxygenation level-dependent or BOLD contrast) as a measure of a hemodynamic response associated with neuronal firing. Utilizing the BOLD signal allows for investigation of region-specific brain activation during performance of a behavioral task. In terms of frontal lobe function, a great degree of variability exists in the aging literature specific to changes in either patterns of or amounts of BOLD activation. This variability is likely due in part to the specific paradigms used, health of the sample, age of the sample, and differences in magnet hardware [25, 26]. One common debate is whether aging is accompanied by increased recruitment of neurons to complete a task relative to younger adults. Some argue that the recruitment of contralateral brain tissue is a sign of compensation to counteract volume loss [27], while others argue that such recruitment is inefficient and therefore detrimental. While this debate is outside the scope of this paper, of most interest to the present examination is the relationship between alterations in fMRI patterns of activation and structural measures of brain volumes.

The purpose of the current study is to use multimodal imaging techniques to examine working memory performance in healthy older adults. Specifically, the investigation examines structural brain GM and WM volume and WM integrity (FA) in relation to functional activation and behavior during performance on a working memory (*N*-Back) task between healthy younger and older adults. Our primary hypothesis is that reduced integrity of white matter and not alterations in cerebral volumes is associated with poorer working memory performance in normal aging.

## 2. Methods

### 2.1. Participants

All participants provided written informed consent, and experimental procedures complied with the code of ethics of the World Medical Association, the University of Illinois Institutional Review Board, and Declaration of Helsinki. Twenty-five healthy younger adults (mean age = 26 years, SEM = 2.04 years; mean education = 16.3 years; SEM = 0.65) and 25 healthy older adults (mean age = 65.9 years, SEM = 2.38 years; mean education = 14.6 years, SEM = 0.73) participated in the study. Although the older adults had a trend for less formal education than the younger adults, this comparison did not reach significance (*P* = 0.08). All were right handed, and none reported cognitive, visual, or motor deficiencies other than the need for corrective lenses. All were native speakers of English. Subjects reported no history of diagnosed neurologic disorder (including, but not limited to Alzheimer's disease, mild cognitive impairment, stroke, Parkinson's disease, traumatic brain injury, or attention deficit disorder), psychiatric disorder (including, but not limited to, major depressive disorder, schizophrenia, or bipolar disorder), or history of substance abuse and/or dependence as defined by the DSM-IV (APA, 1994). Additionally, subjects were excluded if they had any history or current use of medication for either hypertension or hyperlipidemia or had a positive history of cardiac illness.

### 2.2. Procedure

All subjects received practice on each version of the *N*-back working memory task prior to imaging. Following this brief practice, subjects were placed in the MRI and completed four versions of a working memory task described below followed by a high-resolution anatomical study and DTI. 

#### 2.2.1. Functional Imaging Methods: *N*-Back

**Figure 1:**
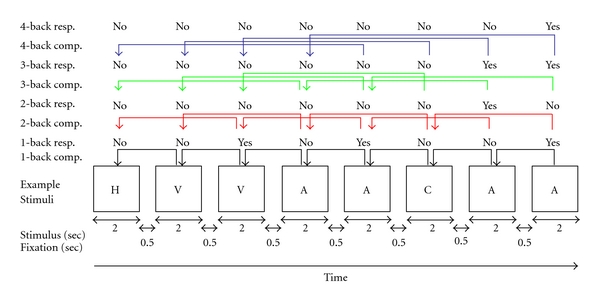
Diagram of sample series of *n*-back stimuli, timing of stimuli (bottom), expected correct responses, and appropriate stimuli to match to under different experimental (*n*-back) conditions.

During the fMRI assessment, subjects performed four versions of a verbal *N*-Back task [28]. Each version involved two conditions—0-back and “*N*”-back (where *N* is either 1, 2, 3, or 4 back). The *N*-Back is a well validated test of working memory (Lezak, 1995) and requires subjects to monitor a series of letters presented on a computer screen and to respond if the letter is identical to the one that immediate proceeded it (1-Back), the one presented two trials back (2-back), three trials back (3-back), or four trials back in the list (4-back). Instructions were first presented for 10 sec and included information on the nature of the task (e.g., whether the task was 0-, 1-, 2-, 3-, or 4-back). For the 0-back conditions, the participants needed to retain in memory a reference target that had been presented in the instructions. Following instructions, a series of 20 letters (trials) were presented. Each letter was presented for 2 sec and was followed by 500 msec of fixation. Participants indicated whether the currently presented letter matched the identity of the trial (reference, 1, 2, 3, and 4 back) target by pressing an MR compatible response switch (MRI*χ* Technologies, Bannockburn, Ill, USA). The 0-back task provides an activation map for short-term memory, eye movements, motor planning, and motor execution. Intermixed with blocks of 0-back were the 1-, 2-, 3-, and 4-back conditions. A forced two-choice speeded response was required on each trial (same and different). An example of the materials, appropriate stimulus comparisons relative to condition and with correct button responses is presented in Figure 1.

#### 2.2.2. Functional Imaging Parameters: *N*-Back

All imaging took place on a 3T MRI (GE, 3T EXCITE 2.0). Functional MRI was performed using a blocked design to optimize spatial localization of function (epiRT, plane = axial, TR = 2500 ms, TE = 25 ms, flip angle 60°, NEX = 1, Bandwidth = 62 kHz, acquisition matrix = 64 × 64, FOV = 20 × 20 cm^2^, slice thickness/ISI = 4/1 mm/mm, slices = 30, volumes = 144).

#### 2.2.3. Structural Imaging Parameters: T1 SPGR

Following functional imaging, anatomical imaging for visualization of full brain anatomy was performed with a three-dimensional inversion recovery prepared spoiled gradient recalled echo (3D IRPrepSPGR) acquisition (reconstructed in three planes =axial, coronal and sagittal, TR = 13.8 ms, TE = 2.7 ms, flip angle = 25 degrees, acquisition matrix = 512 × 192, FOV = 22 × 16 cm^2^, slices = 120, slice thickness/separation = 1.5/0 mm/mm, NEX = 1, Bandwidth = 15.6 kHz, total acquisition time = 5 : 33 minutes).

#### 2.2.4. Diffusion Tensor Imaging Parameters

To characterize white matter integrity, we collected DTI on each subject. The sequence is based on a single-shot EPI pulse sequence with the capability of compensating eddy currents induced by the diffusion gradients via dynamically modifying the imaging gradient waveforms. The diffusion-weighting orientations are designed based on the electrostatic repulsion model proposed by Jones et al. [29] (TR = 5000–6000 ms, TE = minimum (81 ms), *B*-values = 0,750 s/mm^2^, diffusion gradient directions = 27, FOV = 22 cm, Matrix = 128 × 128, slice thickness = 5 mm, gap = 1.5 mm, ramp-sampling = on, NEX = 2, total acquisition time = 5 : 46). 

#### 2.2.5. Functional Imaging Analysis

Analysis on the fMRI data was carried out using Statistical Parametric Mapping (SPM2, Wellcome Department of Imaging Neuroscience, London, UK). Data from each individual subject was initially screened and corrected for head motion. Data with greater than 4 mm of motion (across all tasks) was excluded from further analysis. The functional data (EPI images) were first coregistered with the other functional paradigms and then with the corresponding anatomical images. These coregistered images were then normalized to the Montreal Neurological Institute (MNI) template and spatially smoothed the functional data (FWHM = 8 mm). The preprocessed functional data for each individual was then entered into a general linear model in which the experimental variables (memory load; 1-back, 2-back, 3-back, and 4-back) were evaluated and convolved with the hemodynamic response function (HDF). One significant potential issue in the analysis of functional data for the interpretation of age-related differences is the concurrent finding that normal aging also affects the cerebrovascular system which could affect the neurovascular system [30]. These changes are not believed to affect the shape of the BOLD response or the integrity of this response but do affect the ability to detect a response (signal-to-noise ratio). For this reason, contrasts were calculated between conditions for each subject allowing each subject to serve as their own control. Random effects analyses were conducted on these individual difference maps followed by between-group comparisons.

#### 2.2.6. Structural Imaging Analysis for Quantification of Whole Brain and Regional Volumetric Loss

Manual tracings of prefrontal cortex for each subject were accomplished with the Analyze software package (Mayo Clinic Foundation, Rochester, Minn, USA). Two independent raters produced an interrater reliability of 0.92. We broadly defined the prefrontal cortex using Brodmann's definitions to include the area between the superior rostral sulcus, and inferior rostral sulcus, and dorsally by the anterior cingulate. Given the small subject numbers, we will only present these gross volumes in the present paper. Once drawn, the images were segmented for gray matter, white matter, and cerebrospinal fluid. Total prefrontal volume was extracted for both white and gray matter. 

#### 2.2.7. Diffusion Tensor Imaging Analysis

The 28 diffusion directions, along with the B0 image, were used to calculate FA as the primary indicator of white matter integrity. The images were reconstructed, and FA was calculated, using the program from Johns Hopkins, DTI Studio [31]. The 28 diffusion-weighted image sets were examined for image quality and head movement. Head movement was required to be within one voxel across the image acquisition. Because noise can introduce bias in estimates of the eigenvalues and because noise decreases the signal-to-noise ratio, we applied a background noise level to all subjects prior to calculation of pixelwise FA (background noise = 150 scanner units). It is important to note that the application of this criterion and the noise itself can influence calculation of anisotropy. However, because the analyses focus on differences between groups the bias introduced by this noise floor should not influence group differences. The FA maps were then converted to ANALYSE format and read into SPM2 software for analysis (Wellcome Department of Imaging Neuroscience, London, UK). DTI data from each subject was coregistered with their corresponding T1-weighted anatomic image set (after skull stripping) using a normalized mutual information cost function and trilinear interpolation. Normalization parameters were determined based upon the high-resolution T1 image relative to the MNI template. These normalization parameters were then applied to the FA and eigenvalue images. Each image was visually checked for accuracy after both the coregistration and normalization steps.

#### 2.2.8. Statistical Analysis

In additional to the imaging analyses described above the dependent measures extracted from the imaging data (volumes and fractional anisotropy) were submitted to a mixed design analysis of variance with subject group (younger and older) as the between subjects factor with (df = 1,49). For the *n*-back, percent correct, response latencies, percent of hits and false alarms, and the signal-detection parameter *d*-prime (*d′*) were calculated. The mixed design analysis of variance with participant group as the between subjects factor was performed for each of these measures derived from the behavioral. Because of the sample size, bivariate Pearson correlations were used to examine the relationship between behavioral responses and cerebral tissue volume and integrity. 

## 3. Results

### 3.1. Behavioral Data

**Figure 2:**
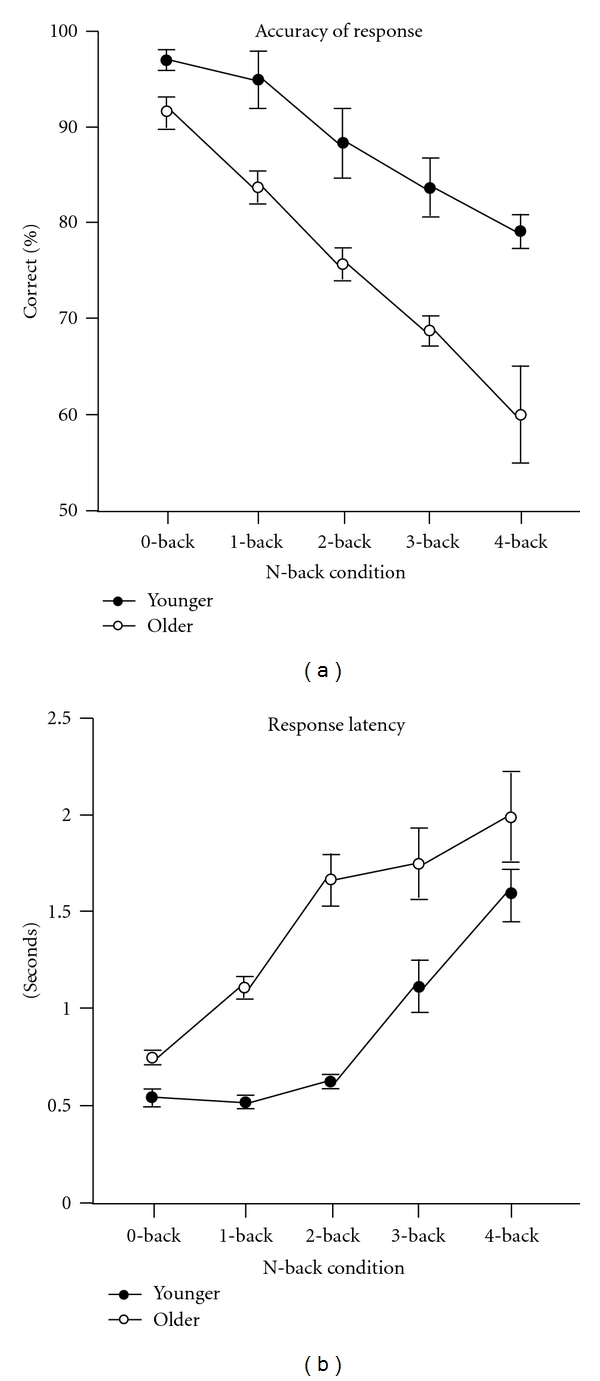
Accuracy (a) and latency (b) for older (open circles) and younger (closed circles) adults collected during fMR data acquisition. Error bars represent 1 SEM.

Behavioral data (i.e., accuracy and reaction time) collected during the fMRI sequences are presented in Figure 2 for both the older and younger adults. First, there was an interaction between age and accuracy (*P* = 0.04) and latency (*P* = 0.02) with older adults showing a greater decrease in accuracy and an increase in latency with increased task difficulty relative to the younger adults. Post-hoc tests of independent means of accuracy within each level of the *N*-Back comparing the young to the older adults showed significant differences at the 0-back (*P* = 0.041), 1-back (*P* = 0.028), 2-back (*P* = 0.048), 3-back (*P* = 0.046), and 4-back (*P* = 0.032). Post hoc comparisons on latency data demonstrated significantly increased latencies for the older adults only at the 0-back (*P* = 0.043) and 1-back (*P* = 0.018) conditions. Because the older adults did not perform better than chance on the 4-back (*P* = 0.15), these imaging data have been excluded from the next set of analyses and results. 

Because of the nature of the *n*-back task and relatively low numbers of trials for which a “match” between target and reference occur signal detection theory was applied to these data. For this, the percent of hits and false alarms were calculated as was *d′*. These data are presented in Table 1. When comparing the performance between younger and older adults using these methods, it becomes clear that the older adults had significantly fewer hits (identifying the target as a target; the only significant (*P* < 0.05) comparison was during the 3- and 4-back) and more false alarms (identifying a nontarget as a target) than the younger adults (all comparisons between groups within each condition *P* < 0.05 with the exception of the 1-back). They also differed on *d′* on all conditions (*P* < 0.05).

### 3.2. Volumetric Measurements

**Figure 3:**
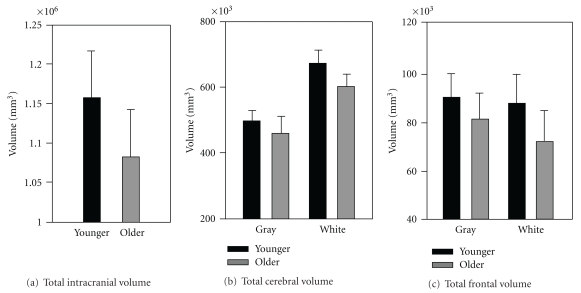
Volumetric data for older relative to younger adults for (a) total intracranial volume, (b) whole brain gray and white cerebral volumes (black bars, young; white bars, older), (c) prefrontal gray and white (black bars, young; white bars, older). Error bars represent 1 SEM.

Volumetric measurements of total intracranial volume, whole brain volumes, and prefrontal volumes for both GM and WM are presented in Figure 3. Differences between younger and older adults on total cranial volume (*P* = 0.09), whole brain GM volume (*P* = 0.071) and prefrontal GM volume (*P* = 0.082) approached, but did not reach, statistical significance. There were, however, significant differences in total WM volume and total prefrontal WM volume, with the older showing smaller tissue volumes than the younger adults (whole brain: *P* = 0.048; prefrontal: *P* = 0.038). 

### 3.3. WM Integrity

**Figure 4:**
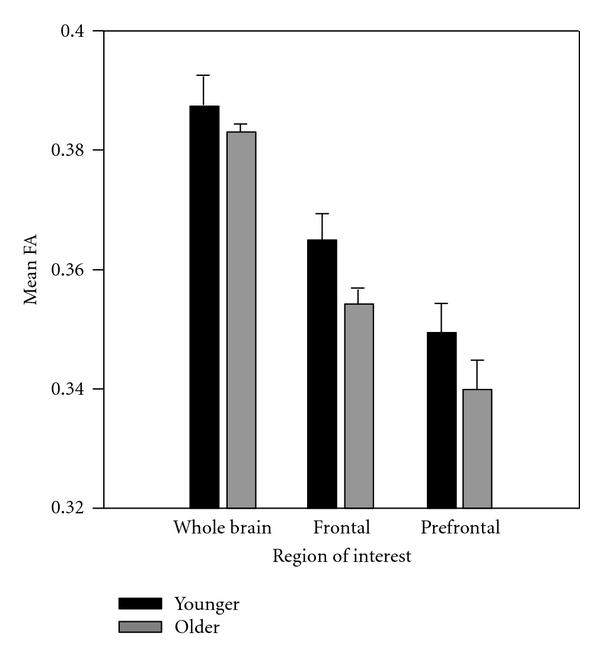
FA for older and younger adults for both frontal and whole brain regions of interest. Error bars represent 1 SEM.

Figure 4 shows whole brain and frontal lobe measurements of FA for the younger and older adults. Compared to younger adults, older adults showed lower whole brain FA values (*P* = 0.018), lower frontal lobe FA values (*P* = 0.038), and significantly lower prefrontal values (*P* = 0.32) indicating lower WM integrity in the older group. 

### 3.4. fMRI Activation Patterns

**Figure 5:**
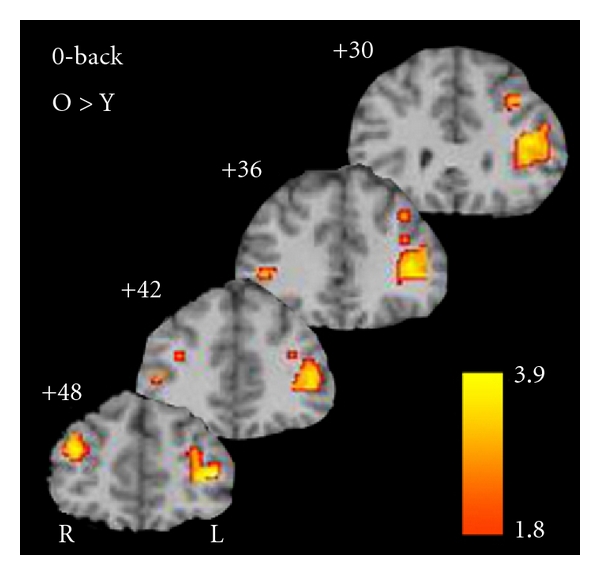
Regions of greater activity for older compared to younger adults on the 0-back. Areas exceeding *P*_corr_ < 0.05 are included.

Comparisons between older and younger adults on the 0-back conditions yielded no significant clusters of activation (*P*_corr_ < 0.05, k = 30) for which the younger adults showed greater activity compared to the older adults. Older adults showed differentially greater activity bilaterally in both dorsolateral prefrontal cortex (DLPFC) and ventrolateral prefrontal cortex (VLPFC) compared to the younger adults (Figure 5).

In light of the group differences in baseline prefrontal cortex activity during the 0-back, the next set of analyses examined age differences as a function of condition (e.g., *n*-back relative to 0-back) in a two-way ANOVA (group × condition (0-back versus 1-, 2-, and 3-back). These analyses address the question of relative increases in activation during parametrically greater memory loads. 

**Figure 6:**
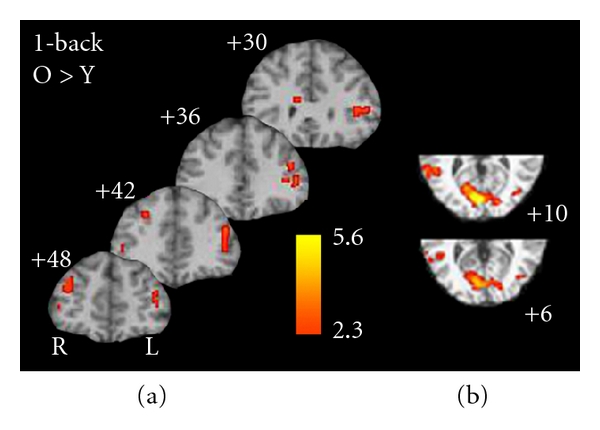
Regions of activation that were differentially greater for older adults on the 1-back (relative to 0-back) compared to younger adults.

**Figure 7:**
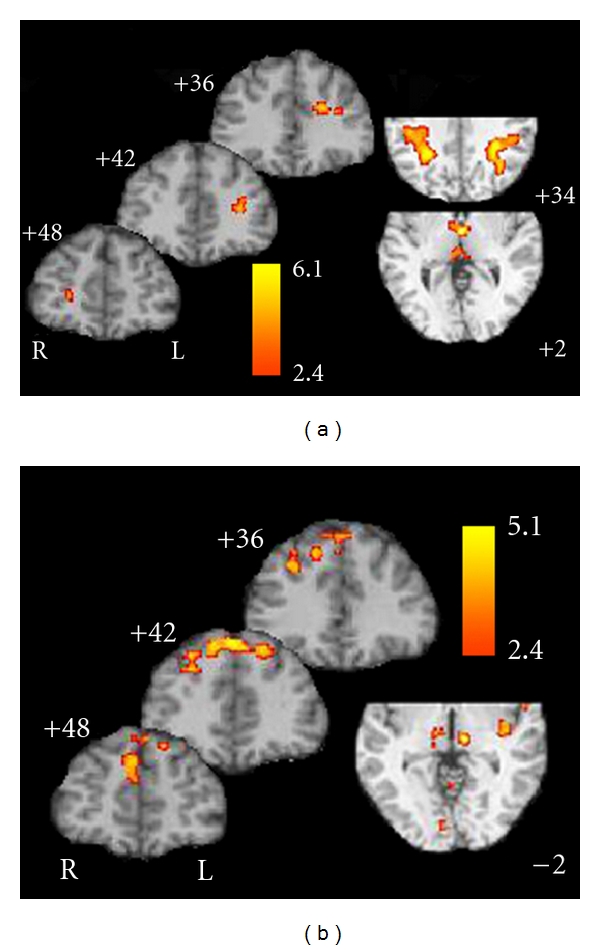
Regions of activation that were differentially greater for younger (a) relative to older adults and those that were differentially greater for older compared to younger adults (b) on the 2-back task.

**Figure 8:**
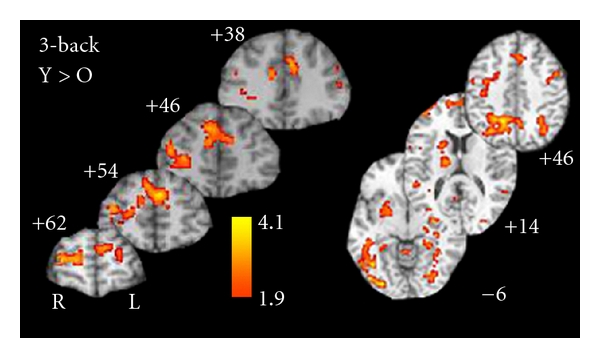
Regions of activation that were differentially greater for younger adults on the 3-back relative to 0-back activation compared to the same contrast in older adults.

As shown in Figure 6, when controlling for baseline activation (0-back), the older adults showed differentially greater activity on the 1 back relative to younger adults especially in prefrontal and ventral-lateral prefrontal regions. The only exceptions to this finding was in primary and higher older visual cortical areas (Figure 6(b)), where the young showed greater activity than the older adults. Additionally, two distinct patterns emerge from the *n*-back data. In the 2-back, the younger adults showed greater activity in deeper frontal structures, the thalamus, and bilaterally in the intraparietal sulci (Figure 7(a)). In contrast, the older adults showed increased activation superior and medially in the frontal lobe. Interestingly, they also showed increase activation in the dorsomedial and pulvinar regions of the thalamus (Figure 7(b)). During the 3-back (Figure 8), the young showed increase activation in a widespread network including the frontal regions, thalamus, basal ganglia, motor regions, intraparietal sulci, and anterior cingulate compared to the older adults. There were no regions which showed increased activation on the 3-back relative to the 0-back for older adults compared to younger adults. 

### 3.5. Structure-Function Correlations

**Figure 9:**
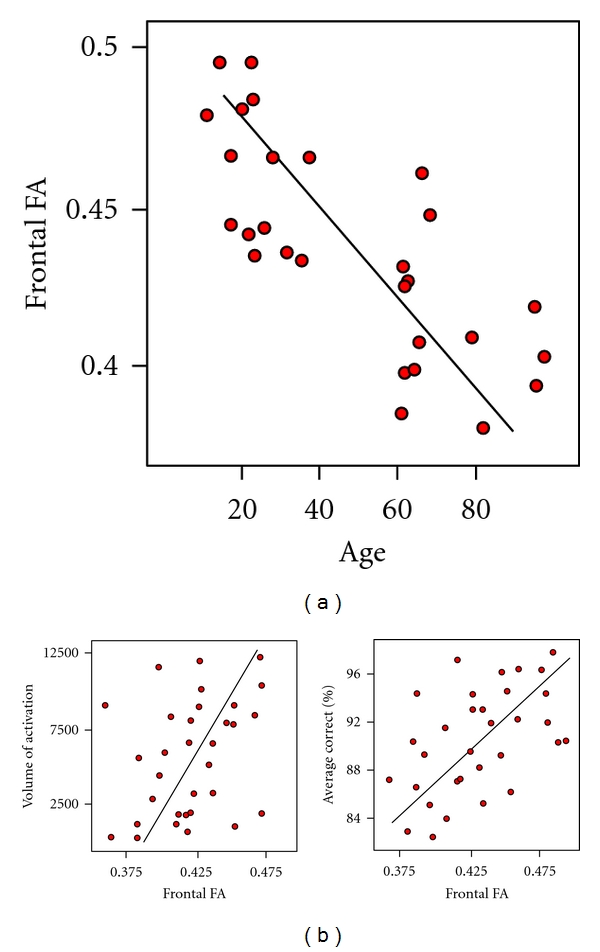
Scatterplots with best-fit lines indicating the relationship between age and FA (a), and FA with volume of activation and behavioral performance (b).

We next conducted a series of correlational analyses to examine structural brain outcomes (e.g., GM volume, WM volume, and WM integrity) in relation to functional activation and behavior during performance of the *n*-back task. For simplicity, accuracy data were collapsed across the 0-, 1-, 2- and 3-back conditions (the 4-back was excluded due to near chance data for the older adults). The results are presented in Figure 9. 

Age was negatively correlated with frontal FA (*r* = −0.69, *P* = 0.001) and frontal lobe FA is positively correlated with the volume of frontal lobe activation (*r* = 0.807, *P* < 0.001) and accuracy (*r* = 0.511, *P* = 0.036). When age was included as a covariate, frontal FA still correlated with fMRI activation (*P* = 0.006) but no longer correlated significantly with performance (although the trend still suggests the same relationship). When the relationship with GM volume was investigated after controlling for age, the only significant relationship was between frontal GM and volume of activation, and this relationship appears to be driven by the 2- and 3- back condition. Interestingly, WM volume did not correlate with performance or volume of activation.

## 4. Discussion

The overall objective of this research was to use multimodal imaging techniques to examine working memory performance in healthy older adults. Two groups of healthy adults (young, 20–30 years of age and older, 60+ years of age) completed four versions of a verbal *N*-Back task (1-back, 2-back, 3-back, and 4-back) while fMRI data were acquired. Additional data was also acquired to characterize integrity of the frontal lobe white matter (diffusion tensor imaging, DTI) and gray matter (inversion recovery spoiled gradient recalled, IRSPGR). With increased age, longer latencies (1-back and 2-back) and decreased accuracy (2-back only) was observed behaviorally. Contrasts between the 1-back and 2-back with a 0-back control task showed the older sample demonstrated increased activation in both dorsal (left) and ventral (left) lateral prefrontal cortical regions for both the 1- and 2-back tasks. Additionally, older adults showed more bilateral activation in prefrontal regions than the younger group. Similar to previous reports, gray matter volumes were negatively correlated with decreased performance (accuracy) on the working memory tasks for the older adults. Findings from Mattay et al. [32] and Cappell et al. [33] show similar patterns of activation in their group of 10 young and 12 older adults. Specifically, Mattay and colleagues showed greater activity in the easier condition in prefrontal cortex for older adults and greater activity in prefrontal cortex during the higher load conditions for younger adults.

Evidence from other functional neuroimaging studies shows altered or disrupted patterns of neuronal function in the prefrontal cortex in elderly adults compared to their younger counterparts [27, 34–37]. Consistent with our findings of recruitment under conditions of lower working memory, memory demand, and reductions in activation under high working memory demand, others have found that the presence or magnitude of recruitment in older adults varies as a function of performance success although these studies are based primarily upon episodic and not working memory. 

Recent neuroimaging findings show modest volumetric loss of both GM and WM during aging. Age-related GM loss is conservatively estimated at somewhere between 3%–5% per decade [2, 38]. However, unlike the linear losses of GM, WM remains largely intact until late-middle-adulthood when volumetric loss occurs in an exponential manner [1, 17] and may decline at a greater rate in frontal regions [39–41]. In the present study, fractional anisotropy was calculated from the DTI data to characterize white matter integrity in the frontal lobes. Our data demonstrate that white matter integrity was stable in younger adults but was reduced for the older adults. Observations of increased neuronal activation both locally (greater volume but in the same region) and bilaterally in older adults in response to increased task difficulty has been interpreted as evidence of neuronal recruitment and support the conclusion that active compensatory processes are observed [27, 42, 43]. In the present study, decreased FA was related to increased baseline activation in the prefrontal cortex offering further evidence of potential compensatory mechanisms. 

In summary, the data presented here demonstrate age-related effects on behavior, white and gray matter volume, white matter integrity, and fMRI activation as a function of working memory task difficulty. Although preliminary and limited by power, the present results begin to integrate previously reported, yet distinct, findings of behavioral, functional, and anatomical correlates of working memory declines in normal aging. Although this study represents only a preliminary attempt at describing these relationships, this hypothesis appears to be supported. However, more discrete analyses of specific white matter tracts and the relationship between these tracts and functional activation are needed to fully describe this relationship and are planned for a larger sample of subjects.

## Figures and Tables

**Table 1:** Percent hits, false alarms, and *d*′ for each condition of the *n*-back within each participant group.

	Condition	Hit rate (%)	False alarm (%)	*d*′
	0-back	96	2	2.69
	1-back	95	10	2.07
Younger	2-back	91	13	1.74
	3-back	85	16	1.44
	4-back	83	15	1.41

	0-back	95	10	2.07
	1-back	94	14	1.86
Older	2-back	88	18	1.48
	3-back	76	21	1.07
	4-back	52	22	0.58
